# The T1‐tetramerisation domain of Kv1.2 rescues expression and preserves function of a truncated NaChBac sodium channel

**DOI:** 10.1002/1873-3468.14279

**Published:** 2022-01-19

**Authors:** Nazzareno D’Avanzo, Andrew J. Miles, Andrew M. Powl, Colin G. Nichols, B.A. Wallace, Andrias O. O’Reilly

**Affiliations:** ^1^ Department of Pharmacology and Physiology Université de Montréal Canada; ^2^ Institute of Structural and Molecular Biology Birkbeck University of London UK; ^3^ Department of Cell Biology and Physiology Center for the Investigation of Membrane Excitability Diseases Washington University School of Medicine USA; ^4^ School of Biological & Environmental Sciences Liverpool John Moores University UK

**Keywords:** channel assembly, chimera, membrane expression, tetramerisation, voltage‐gated channel

## Abstract

Cytoplasmic domains frequently promote functional assembly of multimeric ion channels. To investigate structural determinants of this process, we generated the ‘T1‐chimera’ construct of the NaChBac sodium channel by truncating its C‐terminal domain and splicing the T1‐tetramerisation domain of the Kv1.2 channel to the N terminus. Purified T1‐chimera channels were tetrameric, conducted Na^+^ when reconstituted into proteoliposomes, and were functionally blocked by the drug mibefradil. Both the T1‐chimera and full‐length NaChBac had comparable expression levels in the membrane, whereas a NaChBac mutant lacking a cytoplasmic domain had greatly reduced membrane expression. Our findings support a model whereby bringing the transmembrane regions into close proximity enables their tetramerisation. This phenomenon is found with other channels, and thus, our findings substantiate this as a common assembly mechanism.

## Graphical abstract

This study investigated the influence of the cytoplasmic domain on ion channel assembly. When the C‐terminal coiled‐coil domain of the NaChBac bacterial sodium channel was swapped with an unrelated tetramerisation domain on the N terminus, the chimera expressed robustly in the membrane and produced stable, functional tetrameric channels. This indicates that bringing the transmembrane regions into proximity promotes NaChBac functional assembly.

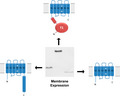

## Abbreviations

**AMP**, ampicillin

**CSA**, camphour sulphonic acid

**CTD**, C‐terminal domain

**LB**, luria broth

**LDS**, lithium dodecyl sulphate

**NMG**, N‐methyl‐D‐glucamine

**PDB**, protein data bank

**POPE**, 1‐palmitoyl‐2‐oleoyl‐3‐phosphatidylethanolamine

**POPG**, 1‐palmitoyl 2‐oleoyl phosphatidylglycerol

**TBS**, tris‐buffered saline

**TM**, transmembrane

Members of the cation channel superfamily are found throughout the domains of life and their activity is crucial for diverse physiological processes including chemotaxis and motility in prokaryotes [1] and action potential generation and propagation in multicellular eukaryotes [2]. A simple tetrameric precursor with two transmembrane helices (TM) per monomer was the most likely progenitor channel, consisting of a pore that permitted ions to cross the membrane while flowing down their electrochemical gradient. Ion channels appear to have then further evolved in a modular fashion, with addition of specialised domains to the core ion‐conducting pore module enabling detection of – and pore‐gating in response to – a variety of stimuli. Most notably, in voltage‐gated channels with six transmembrane helices (6TM), the S1‐S4 helices form voltage‐sensing domains for detecting changes in the transmembrane electric field, whereas helices S5‐S6 form the pore module, with S6 helices lining the pore [3].

Extramembraneous domains have also evolved to contribute to the specialisation and complex regulation of ion channels [4]. In particular, cytoplasmic regions are involved in channel inactivation [5, 6], are sites for phosphorylation [7] and are receptors for the binding of subunits that can modulate channel function [8]. A more fundamental role that cytoplasmic domains perform is promoting channel assembly. With the exception of pseudo‐tetrameric eukaryotic sodium and calcium channels, an oligomerisation step is required for ion channels such as potassium, TRP and prokaryotic 6TM sodium channels to form. While recent high‐resolution structures have revealed the architecture of these channels, the steps involved in assembly remain poorly understood, particularly for processes occurring within the membrane, where the four subunits enclose the central aqueous ion‐conducting pore.

Studies of homo‐tetrameric prokaryotic voltage‐gated sodium channels have yielded important insights into pharmacological and structure–function relationships that are shared with their more complex, pseudo‐tetrameric eukaryotic channel counterparts [9, 10, 11, 12, 13, 14, 15]. The cytoplasmic C‐terminal domain (CTD) of these channels functions as a tetramerisation domain; conserved in the distal end of these CTDs is a ‘a‐d’ heptad repeat sequence motif that is a defining feature of a coiled‐coil structure with a hydrophobic core [16]. A four‐helix bundle coiled‐coil for the CTD was first demonstrated spectroscopically for the NaChBac channel from *Bacillus* *halodurans* [17] and subsequently viewed in crystal structures of NaChBac orthologues [18, 19]. The short ‘neck’ section of the CTD that is contiguous with the S6 helix is flexible and exerts effects on both function and thermosensitivity of these sodium channels [20, 21].

**Fig. 1:**
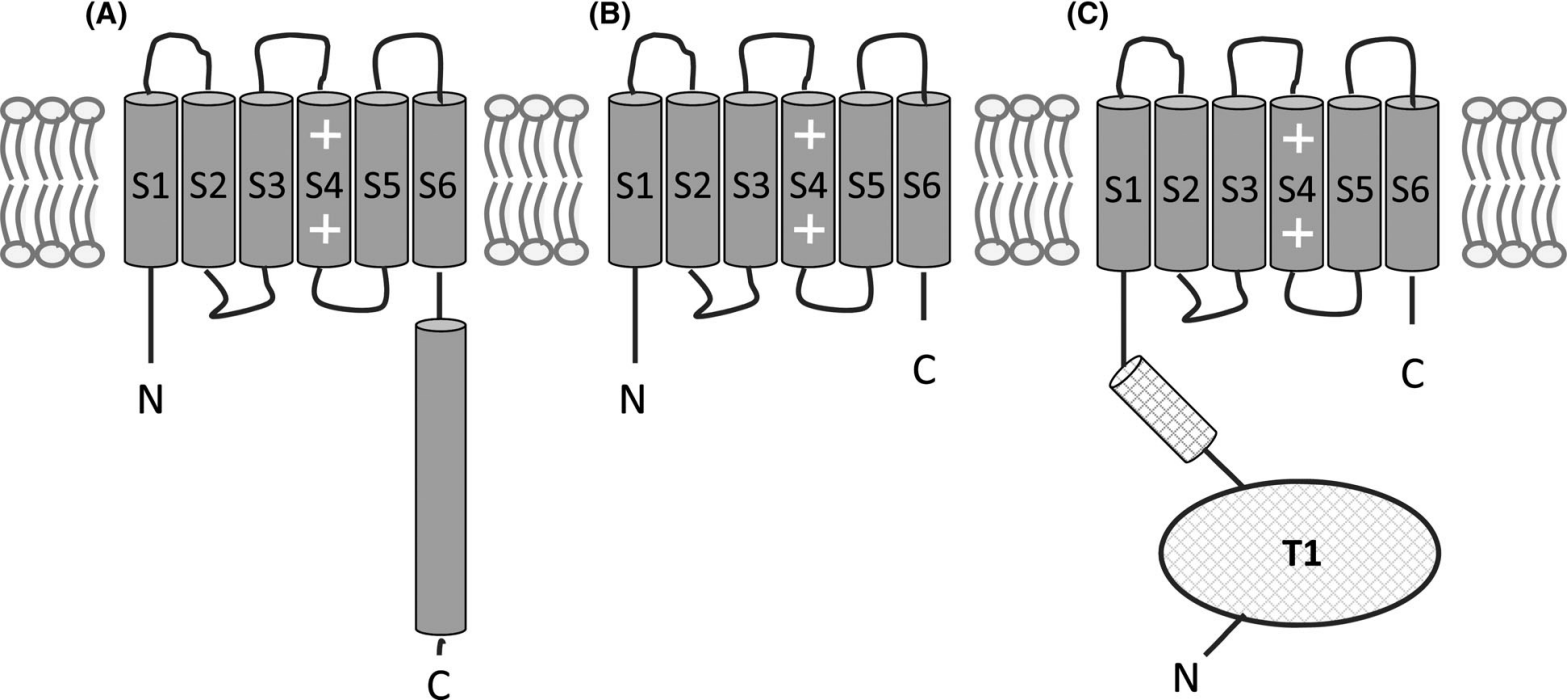
Schematic representation of the NaChBac channel constructs. (A) Full‐length NaChBac, (B) the NaChBac 239Δ C‐terminal deletion mutant and (C) the ‘T1‐chimera’, which has the 239Δ deletion plus the Kv1.2 T1 domain as an N‐terminal extension.

Our previous studies showed that there was a progressive decrease in NaChBac channel membrane expression with truncation of increasing amounts of peptide corresponding to the CTD [17]. In this study, we investigated whether NaChBac expression could be restored by using an alternative tetramerisation domain, which would indicate whether the native CTD promotes channel assembly by simply bringing transmembrane regions into close proximity, as opposed to the CTD imposing specific spatial constraints on these regions. The ‘T1’ tetramerisation domain of the mammalian voltage‐gated 6TM Kv1.2 potassium channel is distinctively different from that of NaChBac. Although this tetramerisation domain is also spatially located below the cytoplasm entrance to the pore [22], it co‐assembles to form a globular fold and is connected to the N terminus of the S1‐S4 voltage sensor by flexible linkers; this arrangement of T1 relative to the transmembrane domain resembles a ‘hanging gondola’ [22, 23]. Here, we generated a ‘T1‐chimera’ channel by truncating the NaChBac CTD and adding the T1‐domain of Kv1.2 to the N terminus and then compared expression of this construct with full‐length NaChBac and the NaChBac 239Δ mutant (Fig. 1). Our studies show that this T1‐chimera forms a tetramer, expresses robustly in the membrane and retains function and pharmacology of the full‐length channel.

## Materials and methods

### Molecular biology

We previously generated pET‐15b plasmids with either the full‐length hexa‐histidine‐tagged NaChBac gene [24] or a truncation mutant with a stop codon introduced after residue 239 (239Δ) [17]. To generate the T1‐chimera of this study, the NaChBac transmembrane domain (residues 9‐239: sequence KQN…ELT) was amplified using forward 5’‐phosphate_CAGAAACAGAACAGTTTCACTAGTAAA‐3’ and reverse 5‐‘GTCGGATCCTCATTACGTTAACTCTGCTTTTTCAACGT‐3’ (BamH1 restriction site underlined) primers. The T1 domain (residues 1‐145: sequence MTV… NEF) of human Kv1.2 was PCR amplified using the forward 5’‐TACGGTCATATGACAGTGGCCACCGGAG‐3’ (NdeI restriction site underlined) and reverse 5’‐ phosphate_AAACTCATTTTCAGGCAGAGGACG‐3’ primers. Both PCR products were electrophoresed and then extracted from 1% agarose, ligated using T4 ligase (New England Biolabs) and used in a further PCR using the T1‐domain forward primer and NaChBac reverse primer. A band corresponding to the correct molecular mass for the chimeric gene was recovered from 1% agarose, digested with NdeI and BamHI (New England Biolabs) and ligated with the pET‐15b vector (predigested with NdeI and BamHI). This subcloning step introduced an N‐terminal hexa‐histidine affinity tag to the T1‐chimera. DH5α chemically competent cells (New England Biolabs) were transformed with ligation mixture (3 µL) and plated overnight at 37 °C on 100 µg·mL^−1^ ampicillin (AMP) agar plates. Plasmids isolated from single colonies by minipreparation were DNA sequenced to verify the gene sequence.

### Cell cultures and western blotting

*E. coli* C41(DE3) cells transformed with pET‐15b plasmid were plated on agar supplemented with 100 µg·mL^−1^ AMP overnight at 37 °C. A single colony was used to inoculate Luria broth + 100 µg·mL^−1^ AMP (LB) and the culture grown overnight at 37 °C was diluted 1 : 100 in fresh LB. Cells were grown at 37 °C on an orbital shaker at 200 r.p.m. until an optimal density of OD_600_ 0.6 was reached, whereupon the temperature was reduced to 22 °C and channel expression was induced with 0.5 mm isopropyl β‐d‐1‐thiogalactopyranoside. Cells were harvested after 8 h by centrifugation at 6000× ***g*** for 20 min at 4 °C.

Expression of channels in the whole cell or membrane fraction was analysed by western blots as described [17]. Aliquots of culture were normalised to an OD_600_ of 2.0 by dilution in LB followed by centrifugation at 6000× ***g*** for 5 min, thus producing pellets with an equivalent amount of cells. A pellet was resuspended and lysed by sonication in lithium dodecyl sulphate (LDS) running buffer (Invitrogen) to produce the whole‐cell lysate. Alternatively, to isolate the membrane fraction, a pellet was resuspended in 20 mm Tris; pH 7.8, 150 mm NaCl (TBS buffer), lysed by sonication and centrifuged at 6000× ***g*** for 5 min, and the supernatant was spun at 100 000× ***g*** for 20 min. The membrane pellet was solubilised in the same volume of LDS running buffer used with the whole‐cell lysate. *S*amples were electrophoresed for 35 min at 200 mV using a 4‐12% NuPAGE Bis‐Tris gel (Invitrogen) and transferred to nitrocellulose membrane using an iBlot system (Invitrogen, Carlsbad, CA, USA). The western blot was probed with an anti‐His antibody conjugated with alkaline phosphatase (Invitrogen Carlsbad, CA, USA) and developed using a Sigmafast BCIP/NBT tablet (Merck KGaA, Darmstadt, Germany).

### Protein purification

Pelleted cells were resuspended (1 g pellet:5 mL buffer) in TBS buffer supplemented with 0.1 mm phenylmethylsulfonyl fluoride and 1 mg·mL^−1^ lysozyme. After stirring for 1 h at room temperature, the solution was supplemented with 10 mm MgSO_4_ and 12 µg·mL^−1^ DNase, stirred for a further 30 min and then lysed by three passes through an EmulsiFlex‐C5 cell disrupter. Unbroken cells were pelleted at 10 000× ***g*** for 45 min, and the supernatant was centrifuged at 200 000× ***g*** for 2 h. Pelleted membrane was resuspended in TBS buffer with 1% dodecyl maltoside (1 g membrane : 20 mL detergent solution), homogenised with Eurostar digital (Ika) apparatus at 2000 r.p.m. for 20 strokes and left gently stirring for 2 h at room temperature. Unsolubilised membranes were pelleted at 20 000× ***g*** for 30 min, and the supernatant was supplemented with 10 mm imidazole and bound with TALON cobalt resin (Clontech Laboratories, Mountain View, CA, USA) (1 g : 20 mL solubilised membranes) with gentle agitation for 8 h at 4 °C. The resin was packed into a 5 mL polypropylene column (Qiagen, Hilden, Germany), washed with TBS, 0.3% Cymal‐5, 10 mm imidazole and channels were eluted with the same buffer supplemented with 250 mm imidazole. Protein was concentrated to ~ 10 mg·mL^−1^ using a Vivaspin concentrator (50 kDa cut‐off) and loaded onto a Superdex 200 10/300 GL (GE Healthcare, Chicago, IL, USA) size‐exclusion column pre‐equilibrated with 20 mm sodium phosphate, 150 mm NaCl, 0.3% Cymal‐5 at a flow‐rate of 0.3 mL·min^−1^ at 4 °C.

### Protein cross‐linking

Protein cross‐linking was carried out as described [25]. 5 µL of 250 mm glutaraldehyde was added to 100 µL of purified T1‐chimera channel to give a 12 mm final glutaraldehyde concentration. The reaction was carried out at 25 ᵒC and aliquots were removed at various intervals and quenched by addition of 1 m Tris; pH 8 to a final concentration of 125 mm. Reducing LDS sample buffer was added to the aliquots and the cross‐linked products were analysed by SDS/PAGE.

### Circular dichroism spectroscopy

CD spectra of the T1‐chimera were measured on an Aviv 62D spectropolarimeter in step scan mode, over a wavelength range of 280 nm to 185 nm, with a data interval of 1 nm and a dwell time of 1 s. CD spectra of full‐length NaChBac were obtained under similar conditions on SRCD beamline CD1 at the ISA facility, University of Aarhus, Denmark. Measurements were made in Supracil quartz cylindrical demountable cells (Hellma) with path lengths of 0.05 cm (T1‐chimera) and 0.024 cm (NaChBac). The protein concentration of the T1 chimera and full‐length NaChBac samples were obtained using a Nanodrop 1000 UV/vis specrophotometer and were 1.35 mg·mL^−1^ and 2 mg·mL^−1^, respectively. Data processing was performed using the CDTool software [26]: three repeat scans of both sample and buffer baseline were averaged and then the averaged baseline subtracted from the averaged sample spectrum. The resultant trace was calibrated against a spectrum of camphour sulphonic acid (CSA) measured prior to the experiment [27] on the respective instruments and then scaled to units of Delta epsilon (Δε) using mean residue weight of 116 Da (T1‐chimera) and 115 Da (NaChBac). Secondary structure analysis was carried out using the DichroWeb analysis server [28]. The values cited are the averaged results obtained using the CONTINLL [29], SELCON3 [30] and CDSSTR [31] algorithms in conjunction with the SMP180 reference dataset [32].

### ^22^Na^+^ flux assay

Experiments were performed as previously described [33, 34, 35]. A 3 : 1 mix of POPE (1‐palmitoyl‐2‐oleoyl‐3‐phosphatidylethanolamine) : POPG (1‐palmitoyl 2‐oleoyl phosphatidylglycerol) (Avanti Polar Lipids, Inc.) was solubilised in buffer A (450 mm NaCl, 10 mm HEPES, 4 mm N‐methyl‐d‐glucamine (NMG), 1 mm EDTA pH 7.5) at 10 mg·mL^−1^. 30‐40 µg of either purified T1‐chimera or full‐length NaChBac channel protein was added to 1 mg of solubilised phospholipids and incubated for 30 min. Liposomes were formed by adding the protein/lipid sample to partially dehydrated Sephadex G‐50 columns presoaked in buffer A through polystyrene columns (Pierce Chemical Co.) and spinning to 1300× ***g***. A second set of partially dehydrated columns containing beads soaked in buffer B (400 mm sorbitol, 10 mm HEPES, 4 mm NMG, 1 mm EDTA pH 7.5) were used to exchange the extraliposomal solution by spinning the sample at 1300× ***g***. To initiate ^22^Na^+^ uptake, 400 µL of buffer B containing ^22^Na^+^ and the indicated concentration of the channel blocker mibefradil (Merck KGaA) was added to each sample. 60 μL aliquots of the liposome uptake reaction taken at 5, 10, 20 and 45 min for the T1‐chimera sample (or 15, 30, 60 and 120 min for the full‐length NaChBac sample) were flowed through a 0.5 mL Dowex cation exchange column charged with NMGH^+^ to remove extraliposomal ^22^Na^+^. These aliquots were mixed with scintillation fluid and counted in a liquid scintillation counter. Fractional flux at 60 min in the presence of mibefradil was determined for each concentration following data subtraction of uptake counts measured at each time point in protein‐free liposomes.

### Homology modelling

An homology model of the T1‐chimera was generated based on two template structures. The 2.7 Å resolution X‐ray crystal structure of the NavAb bacterial sodium channel (PDB code 3RVY) [36] and 2.9 Å structure of the rat Kv1.2 potassium channel (PDB code 3LUT) [37] were retrieved from the Protein Data Bank. The transmembrane domains of NavAb and Kv1.2 were superimposed manually using swiss‐pdbviewer software [38] to generate an equivalent orientation of both structures. Coordinates of residues of the Kv1.2 T1‐tetramerisation domain and NavAb transmembrane domain were saved to a single PDB file. This structural template file was used with a sequence alignment of NaChBac with NavAb produced using Clustal Omega [39] to generate homology models of the T1‐chimera using modeller software [40]. 50 models were produced and the top‐scoring according to the internal scoring function of modeller was submitted to the VADAR webserver [41] to verify the stereochemical validity of the model. A figure was produced using pymol (DeLano Scientific, San Carlos, CA, USA).

## Results

### Design of the T1‐chimera

**Fig. 2:**
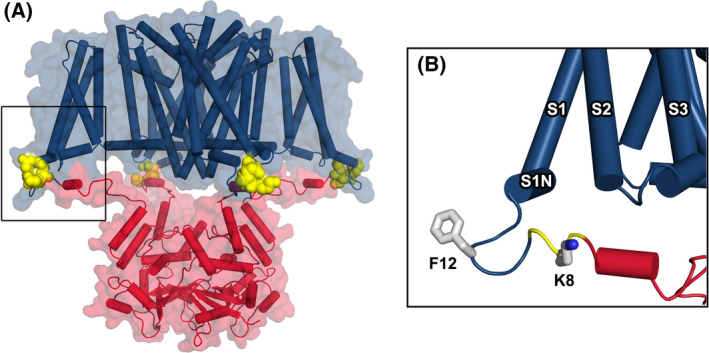
Homology model of the T1‐chimera. (A) The chimeric channel is shown as cartoon and transparent surface with the NaChBac transmembrane domain coloured blue and the Kv1.2 T1 domain coloured red. The transition point between domains occurs at a ‘QKQ’ sequence motif and these residues are shown in space‐fill and coloured yellow. The boxed area in (A) is shown in close‐up in (B) and the side chains of two amino acids at the predicted lipid–cytoplasmic interface are shown as sticks.

The T1‐chimera consists of the transmembrane domain of NaChBac with an N‐terminal extension consisting of the cytoplasmic T1‐tetramerisation domain from Kv1.2 (Fig. 1). The first step in the rational design of this construct was to identify a splice site on the NaChBac N terminus for the T1 domain. A sequence alignment of NaChBac and Kv1.2 showed that there is a ‘QXQ’ sequence motif (where ‘X’ is a positively charged amino acid) that is common to both channels (Fig. S1). Inspection of the Kv1.2 structure shows that QRQ residues are located at the C‐terminal end of the T1 domain on the unstructured cytoplasmic linker that connects to the transmembrane domain. The nine residues that follow QRQ form the short S0 helix of the Kv1.2 voltage sensor. A similar short helix termed 'S1N' is found in the NavAb crystal structure [36]. Sequence alignment of NaChBac and NavAb showed that the homologous S1N region in NaChBac is preceded by the QKQ motif (Fig. S1). Therefore, the QXQ residues of both Kv1.2 and NaChBac appear to be located in an equivalent position, namely at the transition point between cytoplasmic and transmembrane domains. This QXQ motif formed the splice site whereby the seven N‐terminal residues of NaChBac were replaced by the Kv1.2 T1 domain (Fig. 2).

To assess how introduction of the T1 domain might impact the voltage‐sensor structure, the four voltage‐sensor domains of the T1‐chimera model were superimposed upon the same regions of the NavAb template. The root mean square deviation (RMSD) was 0.5‐0.65 Å and visual inspection confirmed close structural alignment, including at the S1N helix. The site of major deviation between NavAb template and model was the region preceding the S1N helix, as this forms a turn in the model to join with the cytoplasmic T1‐domain (Fig. 2B). Located on this turn at the predicted lipid–cytoplasm interface is phenylalanine F12 (numbered according to the NaChBac sequence) and another residue close enough to potentially interact with lipid molecules is K8 of the QKQ motif.

Both the T1 domain of Kv1.2 and the C‐terminal helix‐bundle tetramerisation domain of NaChBac are predicted to share an overlapping site below the cytoplasmic entrance to the pore. Therefore, the second step in design of the T1‐chimera was to identify a suitable truncation point of NaChBac to eliminate potential steric hindrance with the introduced T1 domain. Our modelling indicated that truncation of NaChBac after residue 239 would leave the pore‐lining S6 helices intact while removing the C‐terminal cytoplasmic domain. Specifically, this truncation point occurs 22 residues C‐terminal to the G219 gating‐hinge position in the NaChBac S6 helix [42]. This corresponds to the Kv1.2 structure where 23 residues C‐terminal to the gating‐hinge glycine position in that channel are resolved and shown not to make major contacts with the T1 domain [37]. Fig. 2A shows how the introduced T1 domain can be sterically accommodated below the pore of the T1‐chimera.

### Expression of channel constructs

**Fig. 3:**
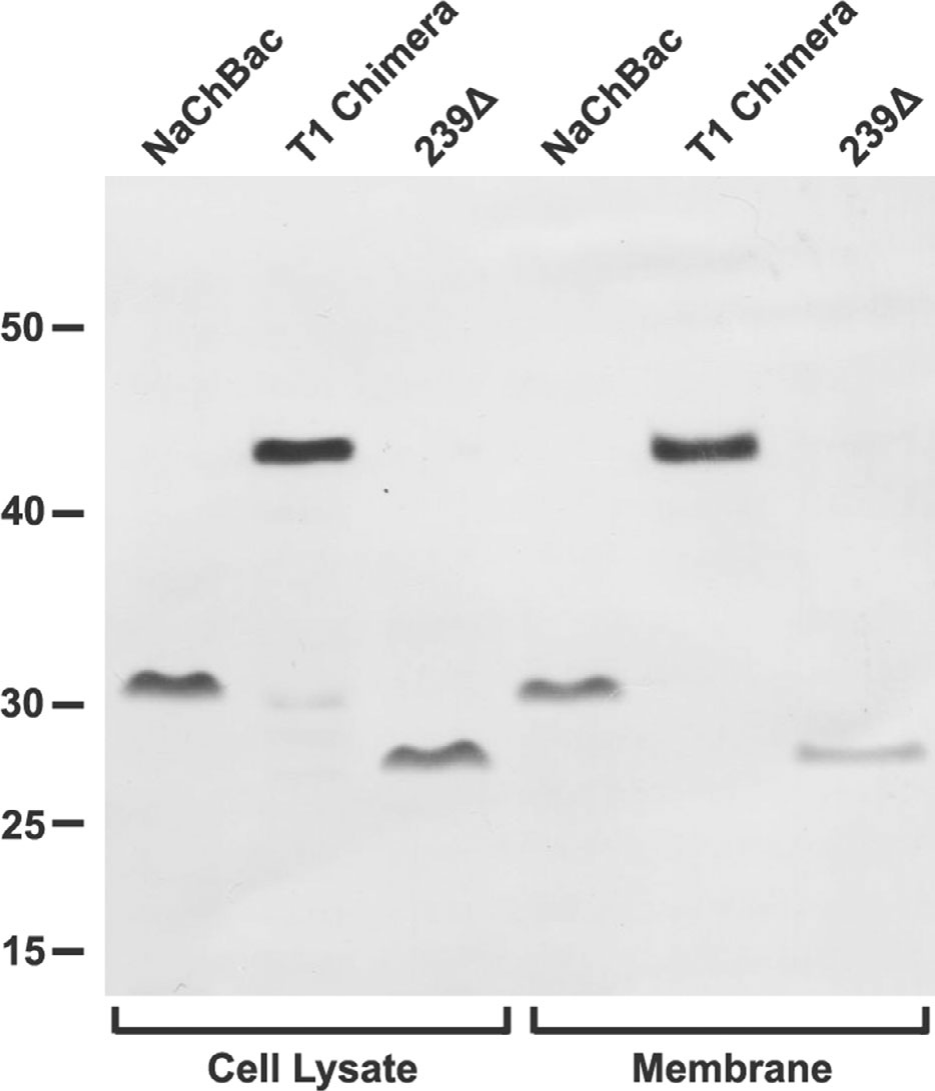
Western blot analyses of channel expression. The cell lysate and membrane fractions were isolated from equivalent masses of *E. coli* cells.

Full‐length NaChBac, the NaChBac 239Δ mutant and the T1‐chimera were expressed in *E*. *coli* under the same conditions. Western blot analysis of cell lysates showed that each protein was expressed at a comparable level (Fig. 3). Comparing the isolated and solubilised membrane fraction from equivalent masses of cells revealed that essentially all full‐length NaChBac and T1‐chimera protein was to be found in the membrane. In contrast, there was notably less of the 239∆ truncated channel in the membrane when compared with the whole‐cell lysate (Fig. 3).

### Purification and glutaraldehyde cross‐linking

**Fig. 4:**
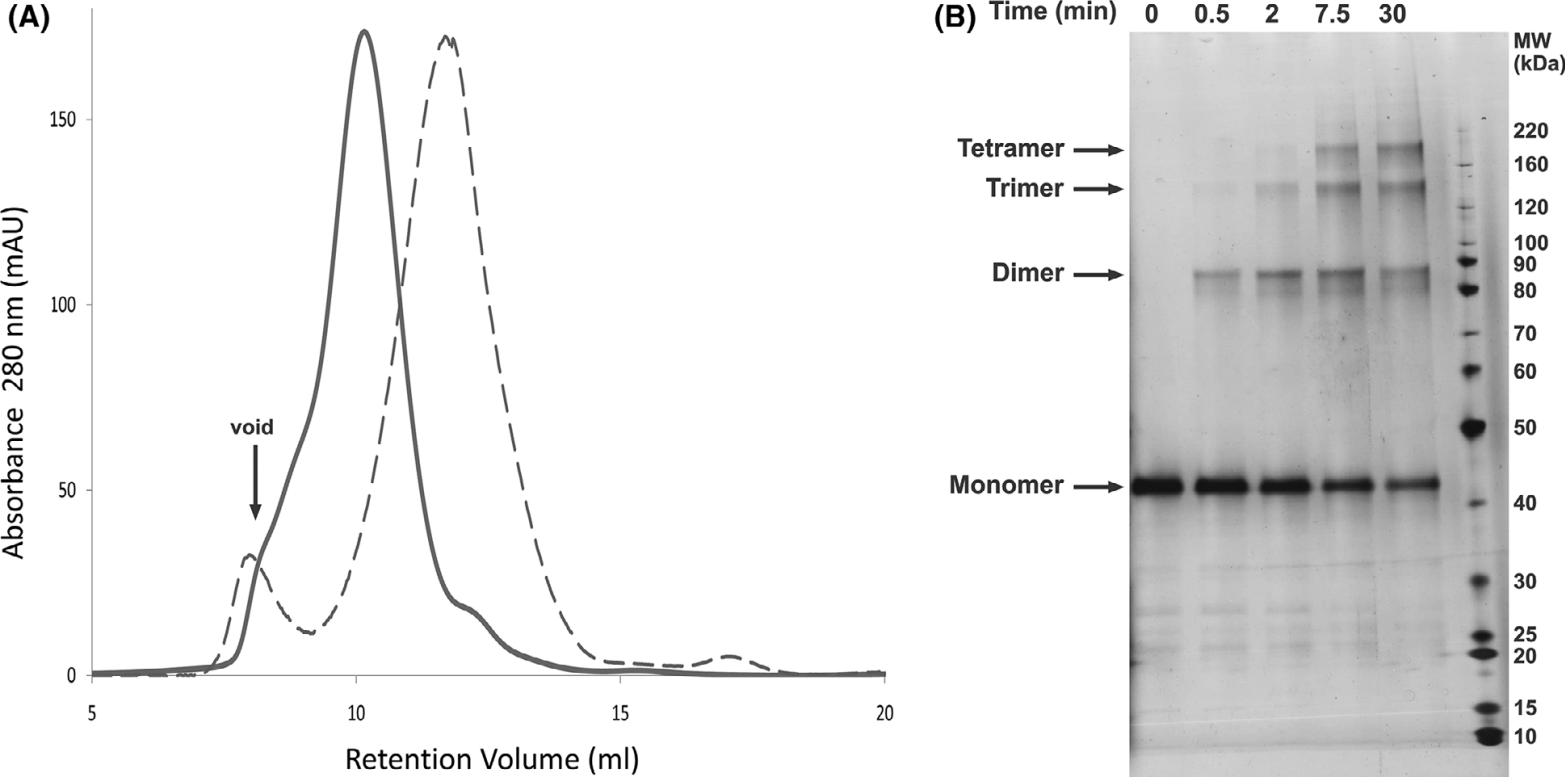
Purification and cross‐linking of the T1‐chimera. (A) Size‐exclusion chromatography profiles of the T1‐chimera (solid line) and full‐length NaChBac (dashed line). (B) 4‐12% SDS/PAGE of glutaraldehyde‐treated T1‐chimera showing the progression of cross‐linking over time.

T1‐chimera channels were purified from detergent‐solubilised membranes by immobilised nickel‐affinity chromatography followed by a size‐exclusion chromatography step (Fig. 4A). The yield of T1‐chimera channels purified to homogeneity (Fig. 4B; time point 0) was 2.3 mg·L^−1^ of cell culture, comparable to the 1.5 mg·L^−1^ yield of full‐length NaChBac obtained under the same conditions, given that the T1‐chimera is larger (184.8 kDa vs 134.4 kDa for tetramers of T1‐chimera and full‐length NaChBac, respectively). Size‐exclusion chromatography reveals a single major elution peak for the T1‐chimera, at a retention volume that is intermediate between the void volume and the full‐length NaChBac (Fig. 4A). This indicates nonaggregate protein that is adopting an oligomerisation state with an overall mass that is larger than that of the full‐length NaChBac tetramer.

Our previous study demonstrated the tetrameric nature of full‐length NaChBac [24], and here, a glutaraldehyde cross‐linking study was undertaken to determine the oligomeric nature of this T1‐chimera (Fig. 4B). At the initial time point, only a single band on the SDS/PAGE gel was present near the expected mass for the T1‐chimera monomer (46.2 kDa). Within 30 secs of glutaraldehyde treatment, a dimeric form was evident, and within 7 min, additional bands corresponding to trimeric and tetrameric forms appeared. No higher oligomers were observed even after 30 min, indicating that the purified T1‐chimera channels were tetrameric.

### Secondary structure of the T1‐chimera

**Fig. 5:**
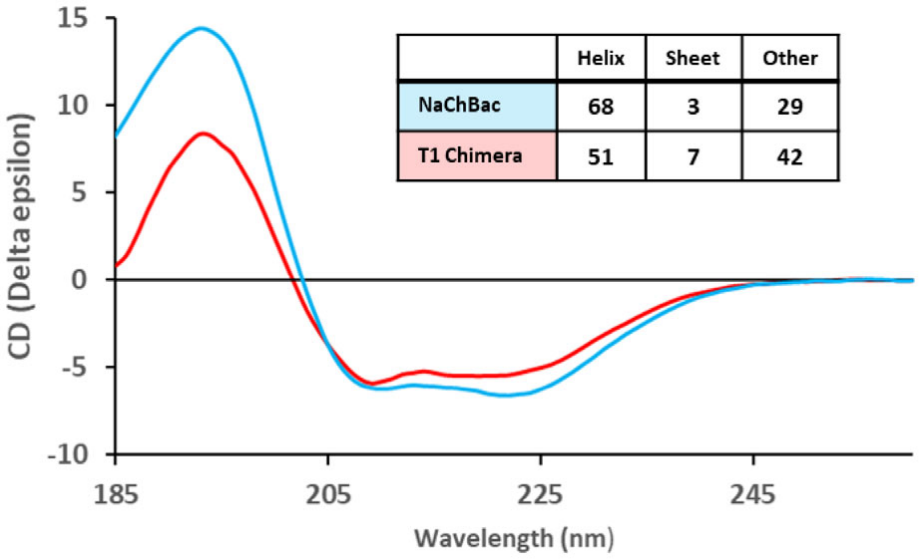
CD spectra of the T1‐chimera (red) and full‐length NaChBac (blue). The table (inset) shows the percentage secondary structure calculated using the average results from CONTINLL, SELCON3 and CDSSTR algorithms in conjunction with the SMP180 dataset, as implemented in the Dichroweb secondary structure analysis server.

We used CD spectroscopy to determine the secondary structure of the T1‐chimera. The CD spectrum of the channel has negative peaks at 222 nm and 209 nm and a positive peak at ~ 193 nm (Fig. 5), which has some resemblance to the spectrum of full‐length NaChBac (Fig. 5). Overall, the CD spectrum of the T1‐chimera is indicative of a folded protein. Determination of secondary structure reveals the T1‐chimera has 51% helicity, whereas the full‐length channel has 68% helicity. This is consistent with known structural features of both proteins as they share the same transmembrane region composed of TM helices yet the CTD of full‐length NaChBac is predicted to be predominantly helical, whereas the T1 domain has two beta strands and numerous loops in addition to short helical segments [37] (Fig. S1).

### The T1‐chimera forms functional channels

**Fig. 6:**
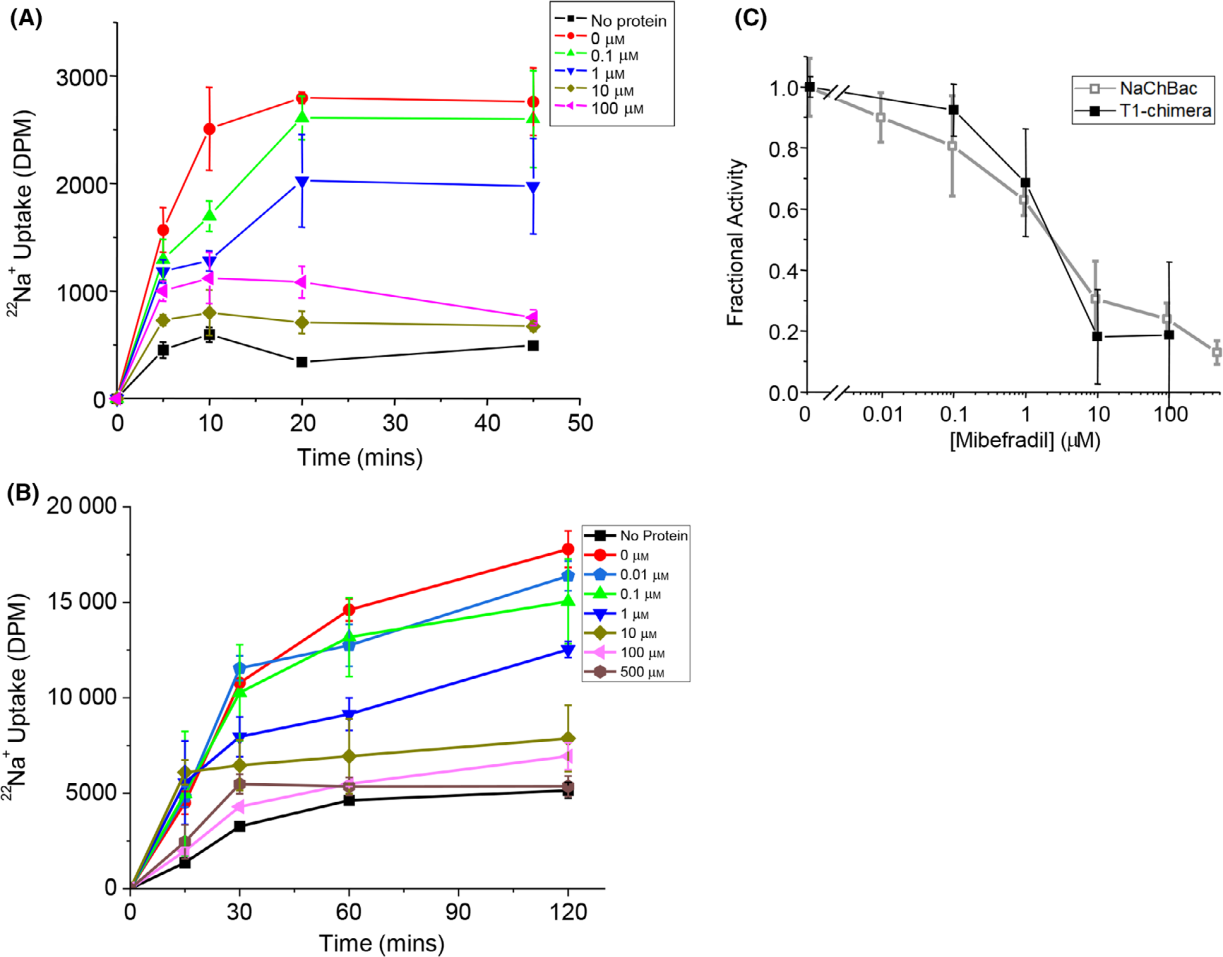
Sodium flux and drug block of the T1‐chimera and full‐length NaChBac. (A) Representative time course experiment of ^22^Na^+^ uptake into liposomes reconstituted with (A) T1‐chimera channels or (B) full‐length NaChBac in the presence of varying concentrations of the channel blocker drug mibefradil. ^22^Na^+^ uptake into protein‐free liposomes is also shown for comparison (squares) (*n* = 6) and was used for background subtraction of uptake. (C) The concentration dependence (mean ± sem) of mibefradil block at 45 min in T1‐chimera channels (filled squares) or at 120 min for NaChBac (open squares) (*n* = 3‐12 for each data point).

Purified T1‐chimera was reconstituted into liposomes formed from 3 : 1 (mol:mol) POPE:POPG for radioactive ^22^Na^+^ flux assays. Protein‐free liposomes did not exhibit significant Na^+^ uptake after 45 min (Fig. 6A). In contrast, ^22^Na^+^ influx was observed in liposomes containing the T1‐chimera and increased over time to reach a maximum intake after 20 min. ^22^Na^+^ influx was also observed when the assays were repeated with full‐length NaChBac (Fig. 6A).

We also tested the effect of the drug mibefradil, which blocks NaChBac [24, 43]. ^22^Na^+^ influx was inhibited in a concentration‐dependent manner for both the T1‐chimera and full‐length NaChBac (Fig. 6A,B), indicating that the observed ^22^Na^+^ uptake was due to conductance through open pores. The inhibition curves for T1‐chimera and full‐length NaChBac channels closely align (Fig. 6C) and the estimated IC_50_ value is 2 µm for both channels.

## Discussion

We generated the T1‐chimeric form of NaChBac to determine whether a structurally unrelated tetramerisation domain, present on the N terminus instead of the C terminus, could provide the same energetic effect to produce functional channels. Our CD and cross‐linking studies show that purified T1‐chimera channels were stable tetramers, while the ion flux results would appear to rule out the possibility that the channels have nonassembled transmembrane regions with disordered chains simply held together by the T1 domain. As found with full‐length NaChBac, the T1‐chimera conducted ^22^Na^+^ and was blocked by the drug mibefradil, thus demonstrating the presence of a functionally intact pore module.

We found previously that protein expression of the NaChBac 239Δ mutant was greatly reduced compared to full‐length NaChBac, but was not entirely eliminated [17] and that this 239Δ mutant could be purified as a tetramer [44]. This indicated that the CTD promotes channel assembly yet is not indispensable to the assembly process, which was also found with other CTD‐truncated NaChBac orthologues [21]. The question remained, however, whether the NaChBac CTD promotes assembly by simply bringing TM regions into close proximity or whether it was acting as a chaperone by promoting specific interactions with, for example, the S5, S4‐S5 or S6 helices. Conceivably, the CTD might orientate and position the contiguous S6 helices to interact optimally for assembly. While we cannot rule out such a chaperoning role, our findings with the T1‐chimera suggest that it is not the major mechanism involved in NaChBac assembly. Instead, the presence of the N‐terminal tetramerisation domain in the T1‐chimera restored expression to full‐length NaChBac levels when compared with NaChBac 239Δ, clearly demonstrating that simply bringing monomers into close proximity sufficed to boost their assembly in the membrane. Equivalent results were obtained by Zerangue et al. when they performed the opposite tetramerisation swap of our study and replaced the T1 domain of Kv1.2 with the four‐helix bundle GCN4‐LI coiled‐coil domain [45]. The percentage of functional Kv1.2 in the membrane was greatly diminished yet not entirely abolished in channels lacking a tetramerisation domain, whereas functional expression was rescued by introducing the alternative tetramerisation domain at the N terminus. Similarly, this unrelated GCN4‐LI coiled‐coil domain restored functional expression of a human hERG potassium channel when it replaced the native CTD [46], further demonstrating that for a diverse range of channels, the specific structure of the cytoplasmic tetramerisation domain is not a crucial factor in TM assembly.

The presence of the T1 domain could potentially affect the structure and function of the TM region of the T1‐chimera in two different ways: first is mechanical perturbation exerted via the T1‐linker upon movement of this cytoplasmic domain. In our model the S1N helix is packed against the S2 helix and may shield the intracellular surface of the voltage sensor as proposed for the NavAb channel [36], whereas a phenylalanine residue (F12) is positioned to bury its side chain in the lipid bilayer and may subsequently resist withdrawal from this hydrophobic environment [47]. Together these structural features may counter‐act a force from the T1‐linker that acts on the N‐terminal region of the voltage‐sensor, thus stabilising this domain. Second, movement of the T1 domain into proximity with the TM region may generate numerous interdomain electrostatic and hydrogen bond interactions, the functional effect of which is difficult to predict. Other potential interactions that merit further study are between the T1‐linker and lipid molecules. R147 (of the QRQ sequence motif) on the Kv1.2 T1‐linker contributes to a binding site for phosphatidylinositol‐4,5‐bisphosphate (PIP_2_) and this interaction affects the voltage dependence of activation to stabilise the closed state [48]. It is therefore possible that K8 (of the QKQ sequence motif) that is present in both the full‐length NaChBac and T1‐chimera may similarly interact with a negatively charged lipid to affect channel function.

The voltage dependence of activation and inactivation was not significantly altered in a 257Δ construct of NaChBac, whereas a more extreme 233Δ truncation produced no functional channels [49]. The truncation point of the T1‐chimera lies between these two positions, and there is reason to believe that truncating the CTD at the 239 position may affect its function. Structures of the full‐length NavMs channel from *Magnetococcus sp*. [19] and a pore‐only construct [20] show that the neck region of this channel can transition between helical and disordered conformations. Specifically, in the full‐length NavMs structure the CTD residues contiguous with the S6 helix remain helical and are followed by a short 6‐residue disordered region that joins with the coiled‐coil. An ‘EEE’ sequence motif on the neck interacts electrostatically with residues on the S4‐S5 linker, and molecular dynamics simulations predict that this stabilises the pore in an open conformation [50]. The neck region of NaChBac contains an equivalent ‘EED’ sequence motif (residues 242‐244), which is eliminated in the 239‐truncated T1‐chimera. Consequently, the pore of the T1‐chimera is predicted to open for shorter periods of time. Nevertheless, the T1‐chimera pore evidently underwent sufficient opening during the time course of the ion flux assays to permit Na^+^ conductance and ingress of the pore‐blocker mibefradil.

As a pore‐blocking drug, mibefradil is likely to form binding contacts simultaneously with more than one of the S6 helices lining the pore [51] and its block of both full‐length NaChBac and the T1‐chimera at an equivalent concentration suggests identical pore structures, thus providing further evidence that the TM regions of these two channels have undergone similar tetramerisation. The mibefradil IC_50_ value is an order of magnitude lower (2 vs 22 µm) than found by Ren and colleagues in their voltage‐clamp study of full‐length NaChBac [43] and a number of factors may account for this difference. First, our liposomes were formed from a mixture of two lipids, which contrasts with the more complex lipid composition found in a mammalian cell plasma membrane and this difference may affect drug partitioning into the bilayer and therefore the available concentration of mibefradil to block channels. Second, the transmembrane voltage and its effect on a channel’s functional state can affect ligand potency and use‐dependent effects on prokaryotic sodium channels have been observed with both the agonist batrachotoxin [15] and antagonist µ‐conotoxin PIIIA [14]. A membrane potential is expected to exist across the proteoliposome bilayer due to the difference in ion concentrations between the extra‐ and intra‐liposomal solutions and this voltage would change over time with ^22^Na^+^ influx, thus producing a dynamic condition that contrasts with the applied transmembrane potential during the voltage‐clamp study [43]. Third, whereas all NaChBac channels expressed in a cell will be in the same orientation, the purified then reconstituted channels of our study are likely to be randomly oriented in the liposomes. This does not pose an issue with our interpretation since we are studying channel‐mediated electrodiffusive permeation rather than directional ion pumping. Nevertheless, channels oriented in the direction where the “physiological” extracellular face of the channel experiences a positive membrane potential would be expected to activate more readily compared with channels oriented in the opposite direction. Voltage‐clamp studies could resolve on the millisecond/second timescale how mibefradil interacts differently between these two populations of channels with ‘orientation‐dependent’ functional transitions but during the minutes‐long flux assay mibefradil presumably had sufficient time to cross the lipid bilayer and target all channels, thus duration of the experiment is a fourth major difference between our study and that of Ren et al [43].

The Na_V_Sp1 sodium channel from *Silicibacter pomeroyi* exhibits considerable thermosensitivity [21], as the voltage dependence of activation shifts by −35 mV with an increase in temperature from 18 to 35 °C, which is almost double the shift found for NaChBac over an equivalent temperature range (20–37 °C) [52]. Thermosensitivity of Na_V_Sp1 is eliminated when the CTD is truncated, identifying this region as an effective thermosensor [21]. Furthermore, mutation of the neck ‘AED’ residues (equivalent to the ‘EED’ sequence motif in NaChBac) to glycine also eliminates this temperature‐dependent voltage‐shift and it is proposed that temperature‐dependent structural rearrangements in the neck region are molecular determinants of thermosensitivity in Na_V_Sp1 and other orthologues [21]. Our previous synchrotron radiation CD studies have shown that the TM region of NaChBac is considerably more thermostable than the CTD, with the neck region predicted to have a disordered secondary structure [17, 44]. As discussed, the neck of the T1‐chimera is dissected and so this channel is expected to have decreased thermosensitivity compared to full‐length NaChBac. Resilience to temperature effects could be a desirable feature in the context of ion channel engineering efforts to generate constructs with tweaked or even novel properties [53]. To speculate about one such novel application, the T1‐chimera could be co‐expressed with a Kvβ oxido‐reductase enzyme, which binds with the T1 domain to modify Kv1.2 function *in vivo* according to the redox potential of the cell [8, 54]. If a co‐expressed Kvβ subunit did indeed confer the T1‐chimera with sensitivity to pyridine nucleotides, then this construct could substitute for Kv1.2 when a Na^+^‐selective current is the desired outcome in response to a change in cellular conditions.

In conclusion, our findings support a model of NaChBac assembly whereby the cytoplasmic tetramerisation domain simply acts to promote assembly by effectively increasing the local concentration of monomers. This allows the TM sections to encounter each other, but evidently the molecular machinery for TM assembly resides within this TM domain. Our domain‐swapped T1‐chimera provides further evidence for the modular nature of ion channels and represents a potential addition to the growing repertoire of engineered ion channels with unconventional properties.

## Author contributions

AOR designed the project, modelled the T1‐chimera and performed the membrane expression study. AP and AOR grew bacterial cultures. AOR purified protein and performed the cross‐linking study. ND prepared proteoliposomes and collected and analysed the ion flux data. AM collected and analysed the CD data. BAW and CGN helped to supervise the project. AOR wrote the manuscript and revisions were provided by all co‐authors.

## Data Availability

The CD spectra have been deposited in the Protein Circular Dichroism Data Bank (PCDDB) [55] under the codes CD0006395000 and CD0006395001. The other data that support the findings of this study are available from the corresponding author [a.o.oreilly@ljmu.ac.uk] upon reasonable request.
