# Supplementary material for: The T1‐tetramerisation domain of Kv1.2 rescues expression and preserves function of a truncated NaChBac sodium channel
Source: FEBS Lett. 2022 Jan 19;596(6):772–83. doi: 10.1002/1873-3468.14279 (PMC9303580; doi:10.1002/1873-3468.14279)
Supplement: Supplementary file 1 [file FEB2-596-772-s001.pdf]

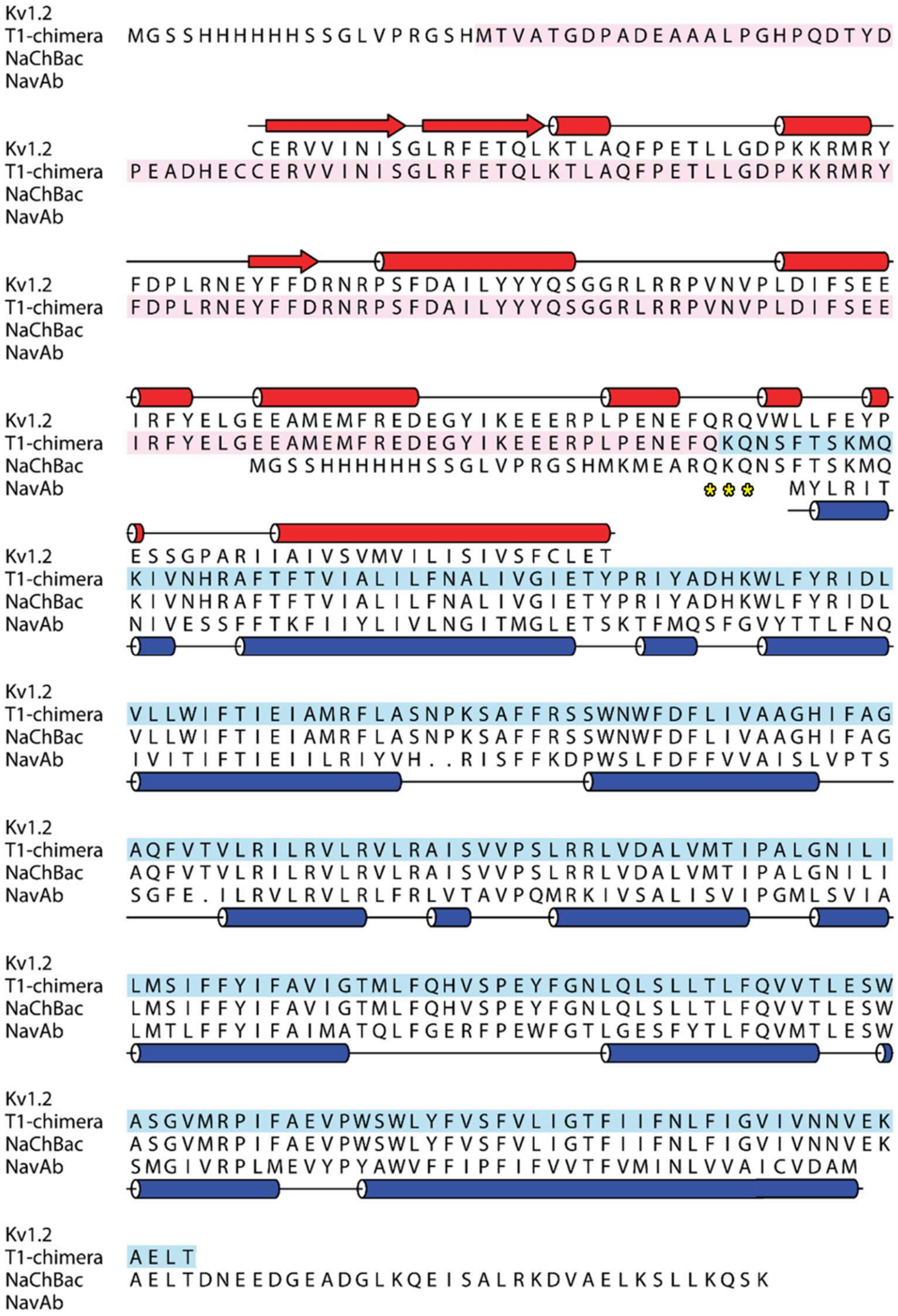

Supplementary Fig. 1. Sequence alignment and secondary structure of channels. 'Kv1.2' is the sequence of the rat Shaker potassium channel resolved in crystal structure (PDB code 3LUT); red cartoons show the T1-domain secondary structure. 'NavAb' is the sequence of the *Arcobacter butzleri* sodium channel resolved in the crystal structure (PDB code 3RVY); blue cartoons show the secondary structure of its transmembrane domain. 'NaChBac' and 'T1-chimera' are the sequences of constructs expressed in this study. 'T1-chimera' is shaded according to cytoplasmic T1-domain (pink) and transmembrane domain (blue). Yellow stars show the QXQ motif (where 'X' is a positively-charged amino acid) that is the cross-over splice site for the T1-chimera.
